# Gambling formats, involvement, and problem gambling: which types of gambling are more risky?

**DOI:** 10.1186/s12889-020-08822-2

**Published:** 2020-05-18

**Authors:** Alissa Mazar, Martha Zorn, Nozipho Becker, Rachel A. Volberg

**Affiliations:** 1 Research project manager and research associate of the Social and Economic Impacts of Gambling in Massachusetts project, University of Massachusetts Amherst, School of Public Health and Health Sciences, 416 Arnold House, 715 North Pleasant Street, Amherst, MA 01003-9304 USA; 2 Data manager of the Social and Economic Impacts of Gambling in Massachusetts project, University of Massachusetts Amherst, School of Public Health and Health Sciences, 416 Arnold House, 715 North Pleasant Street, Amherst, MA 01003-9304 USA; 3 Research assistant of the Social and Economic Impacts of Gambling in Massachusetts project, University of Massachusetts Amherst, School of Public Health and Health Sciences, 100 Holdsworth Way, Amherst, MA 01003-9282 USA; 4 Research Professor and Principal Investigator of the Social and Economic Impacts of Gambling in Massachusetts project, University of Massachusetts Amherst, School of Public Health and Health Sciences, 418 Arnold House, 715 North Pleasant Street, Amherst, MA 01003-9304 USA

**Keywords:** Problem gambling, Gambling formats, Risk, Gambling regulation, Prevention and treatment

## Abstract

**Background:**

The recognition of problem gambling as a public health issue has increased as the availability of gambling expands. Research has found that some formats of gambling are more closely linked to problem gambling than others. Conflicting evidence, however, has emerged, suggesting that the most important consideration is involvement (i.e., number of gambling formats an individual participates in). This debate has important implications for the regulation of gambling formats and for the allocation of problem gambling prevention and treatment services.

**Method:**

Analyses utilized the Baseline General Population Survey (BGPS) and the Baseline Online Panel Survey (BOPS) of Massachusettscollected in 2013–2014. The BGPS contains a representative sample of 9523 Massachusetts adults and the BOPS contains a sample of 5046 Massachusetts adults. All participants were administered the same comprehensive survey of their past year gambling behavior and problem gambling symptomology. Only those who gambled regularly in the past 12 months (*n* = 5852) were included. The Problem and Pathological Gambling Measure was used to classify gambling behavior. Within the sample, there were 446 problem gamblers. We assessed: 1) whether some gambling formats are more related to problem gambling; 2) whether problem gambling is positively related to high involvement in gambling; 3) the relationship between involvement in gambling and intensity of gambling; and 4) whether gambling formats mediate the relationship between gambling involvement and problem gambling.

**Results:**

Groups of monthly gamblers participating in casino gambling, bingo, and sports betting contained a higher proportion of problem gamblers. High gambling involvement was also positively associated with problem gambling; however, a large minority of gamblers experienced problems when engaging in only one or two forms of gambling. Gambling involvement was also positively associated with intensity of gambling. Therefore, intensity of gambling may be partly driving the relationship between involvement and problem gambling. Specific gambling formats mediated the relationship between involvement and problem gambling.

**Conclusions:**

The gambling format an individual participates in is connected to whether an individual is likely to experience problem gambling. We also found that the level of involvement (and its relationship to intensity) may affect the likelihood that an individual will experience problematic gambling behavior. Ultimately, the type of gambling format an individual partakes in does mediate the relationship between problem gambling and involvement. In Massachusetts, participating in casino gambling was more closely associated with problem gambling than other formats across all levels of involvement.

## Background

As governments expand the availability of gambling options to their populations, there has been an increasing interest in problem gambling as a public health issue [1, 2]. Researchers have sought to understand whether particular forms of gambling are more ‘risky’ or conducive of problem gambling behavior than others in an effort to inform gambling regulation and problem gambling prevention and treatment services [3]. For example, individuals who participate in casino gambling (which includes electronic gambling machines [slot machines] and table games) are more likely to experience problematic gambling behavior compared to individuals playing large jackpot lottery games [4]. Demonstrating the discriminative differences between gambling formats and gambling behavior has important policy implications as new forms of gambling are legalized and their availability expands. Recently, however, research has presented conflicting evidence as to whether and *how much* the type of gambling format matters in relation to the likelihood of developing a gambling problem. Indeed, while many argue that some gambling formats are more harmful [5, 6], others suggest that a more critical factor is *involvement* (i.e., number of gambling formats in which an individual engages) [7].

### Gambling formats and problem gambling

Different gambling formats have different structural characteristics that affect the likelihood of an individual who gambles developing a gambling problem [8, 9]. Gamblers are also motivated by the sort of experience they are seeking, which then influences the form of gambling they choose to participate in and affects the likelihood of experiencing a gambling problem [10–12]. For instance, traditional lotteries—as distinct from daily or instant lottery games—allow an individual to wager a small stake for a chance to win a large amount of money and are based on complete chance. Sports betting, in contrast, contains an element of skill, which may influence the outcome while the amount wagered can vary. Slot machines (or electronic gambling machines [EGMs]), alternatively, allow for continuous, rapid play where the individual can engage for long periods of time. Some researchers suggest that EGM play is particularly problematic as it may create a dissociative state of mind or “dark flow” [13].

Population studies have found that problem gambling rates are particularly high among those who engage in certain gambling formats. For instance, Binde [14], examining 18 national prevalence surveys of mostly European countries, found that interactive internet gambling, casino gambling, and EGMs were often associated with problem gambling while sports pools, bingo, horse betting, and sports betting tend to be moderately associated with problem gambling. MacLaren [4], performing a meta-analysis of Canada’s legal gambling industry, found that video lottery terminals (i.e., EGMs located in bars) were the gambling format most closely associated with problem and pathological gambling in Canada. Using Swedish data, Binde, Romild, and Volberg [15] found that EGMs, casino gambling, bingo, and poker were closely related to problem gambling.

Studies have also examined the relationship between gambling formats and problem gambling in clinical populations and in samples of individuals experiencing gambling problems. In a study based on 78 individuals diagnosed with pathological gambling in the U.S., Grant and Kim [16] found that slot machines, cards, and blackjack were the most popular forms of gambling. In another study based on individuals seeking treatment for pathological gambling in the U.S., Stea, Hodgins, and Fung [17] found that the gambling formats that caused major problems for these individuals were video lottery terminals, slot machines, casino games, and lotteries.

### Gambling involvement, intensity, and problem gambling

Involvement is defined as the number of gambling formats in which an individual participates. High involvement in gambling is positively related to problem gambling [12, 18–20]. Individuals who participate in many types of gambling formats (i.e., high involvement) are more likely to find some form(s) of gambling that they become enamored with which then increases the risk of developing a gambling problem [21].

Some analyses have suggested that the relationship between gambling formats and problem gambling is no longer significant or significantly decreases when controlling for involvement [7, 22, 23]. Including number of gambling formats in a multivariate model, however, has significant limitations in discriminating whether particular gambling formats are more or less harmful. This is due to the fact that extensive involvement in several types of gambling is a major aspect of problematic gambling behavior. This is why gambling involvement is not typically used as a predictor. This is also why number of gambling formats tends to be the strongest predictor of problem gambling when used in a model. Indeed, other variables will likely not add much discriminative value when an aspect of a disorder—i.e., involvement—is used to predict the disorder in a model. It is worth noting that the results of regression analyses in studies that statistically control for involvement may be affected by the inherent collinearity between variables since the involvement measure is typically the sum of the variables measuring participation in individual forms of gambling. It is also worth noting that while the involvement hypothesis initially looked only at breadth of involvement, as measured by number of formats engaged in, the hypothesis has been extended to also look at depth of involvement, as measured by frequency of engagement [15].

To bring clarity to the debate between the importance of gambling formats versus involvement in gambling in relation to problem gambling, it is key to tease out the importance of ‘intensity.’ Intensity is the amount of time or money spent gambling. Commonsensically, intensity of gambling is closely related to problem gambling and the relationship between high involvement and problem gambling may be the result of high involvement capturing high intensity. Therefore, intensity may be a more direct measure of problem gambling. Using Swedish data from the first wave of a longitudinal study, Binde, Romild, and Volberg [15], explored the relationship between problem gambling, forms of gambling, gambling involvement, and gambling intensity. These analyses found a strong relationship between involvement and intensity. In addition, Binde, Romild, and Volberg [15] found that, while many individuals experiencing a gambling problem regularly participate in multiple forms of gambling, half of the individuals experiencing a gambling problem in their Swedish study participated regularly in only one or two forms of gambling. These researchers conclude that some forms of gambling are more closely associated with problem gambling than other forms.

This article seeks to further elaborate understanding of the relationship between problem gambling, forms of gambling, gambling involvement, and gambling intensity. We utilize a combination of two Massachusetts datasets to increase the number of available individuals experiencing gambling problems for assessment. These datasets represent the most recent data currently available to assess problem gambling and gambling behavior at a population level in North America.

### Hypotheses

We propose and test the following hypotheses:

- H1: Problem gambling is more closely related to some gambling formats.
- H2: Problem gambling is positively related to high involvement in gambling.
- H3: Involvement in gambling is positively related to intensity of gambling.
- H4:Gambling format mediates the relationship between involvement and problem gambling.

## Methods

### Data collection

Analyses are based on data collected from the Baseline General Population Survey (BGPS) and the Baseline Online Panel Survey (BOPS) of Massachusetts. Utilizing address-based sampling, the BGPS contains a representative sample of 9523 Massachusetts adults (18 years and older). These participants completed a comprehensive survey of their past year gambling behavior and problem gambling symptomology. Data collection was performed by NORC at the University of Chicago. The adult with the most recent birthday was selected as the survey respondent within each sampled dwelling unit. Participants were able to complete the BGPS online, via a paper-and-pencil survey, or by telephone. Data collection was from September 2013 to May 2014. The response rate (AAPOR RR3) was 36.6%.

Data collection for the Baseline Online Panel Survey (BOPS) was conducted by Ipsos Public Affairs. This survey also assessed the gambling behavior of Massachusetts adults. BOPS data collection was from October 2013 to March 2014, which coincided with when data collection for the BGPS was taking place. Ipsos emailed a stratified sample of Massachusetts participants by age, gender, and region. These stratified groups were proportional to the rates reported by the U.S. Census. Until at least 5000 surveys were completed, Ipsos drew additional samples. In the process, Ipsos utilized Massachusetts online panel members from seven partner vendors to supplement their own online panel sample. Initially, 26,913 people were enrolled in the BOPS. However, 18,580 were not eligible (i.e., residing out of state), 2946 did not complete the survey, 293 surveys were not used because of a full gender and age quota, and 48 were eliminated because of poor data quality. A total of 5046 completed surveys were obtained.

The BOPS questionnaire was the same questionnaire used in theBGPS. Past year frequency of participation in eight major forms of gambling was used to examine gambling participation. These were: (1) lottery tickets; (2) instant tickets or pull tabs; (3) daily lottery games; (4) raffle tickets; (5) betting money on sporting events (i.e., sports pools, horse racing, etc.); (6) bingo; (7) casino, racino, or slots parlor outside of Massachusetts; or (8) private betting.

Questions about casino, racino, and slots parlor gambling outside of Massachusetts were included to assess the level of casino gambling among adult Massachusetts residents prior to the availability of casino gambling in the Commonwealth. Information about specific games played at out-of-state casinos was not collected. However, the majority of individuals who had gambled at a casino, racino, or slots parlor in the past year in both the BGPS and the BOPS had done so at the full-service casinos in nearby Connecticut and Rhode Island. The games at the Connecticut and Rhode Island casinos include several thousand EGMs and several hundred table games at each of the four properties along with sports betting, horserace betting, bingo, and keno drawings. In the U.S., EGMs account for between 65% and 80% of casino revenues [24].

Both surveys and the data collection protocols were reviewed and approved by the University of Massachusetts Amherst Institutional Review Board. See Volberg et al. [25] and Williams et al. [26] for a full technical discussion of both the BGPS and BOPS methodologies.

### Measures

These analyses only include Massachusetts residents who have gambled regularly in the past 12 months (*n* = 5852) on at least one of eight major forms of gambling. The Problem and Pathological Gambling Measure (PPGM) was used to determine the survey participants’ problem gambling status [27]. The PPGM is a 14-item assessment with questions organized into three sections: Problems (7 questions), Impaired Control (4 questions), and Other Issues (3 questions). The PPGM employs a 12-month timeframe. This measurement tool also appreciates that gambling behavior exists on a continuum and recognizes four groups of individuals based on their responses (i.e., non-gambler, recreational gambler, at-risk gambler, problem/pathological gambler). In both clinical and population-level settings, the PPGM has been field tested and refined [27]. There were 446 PPGM-designated individuals experiencing gambling problems or more severe pathological gambling within the sample.

These analyses present findings for monthly (i.e., regular) participation since this level of participation is characteristic of problem gambling. Monthly or more frequent involvement was a variable derived from the highest frequency of participation in any major gambling format. Intensity was measured by money spent on gambling and frequency of gambling (as a proxy for time spent gambling).

Participants were asked to report how much money they spent in a typical month for each gambling type. Measures were created to estimate yearly expenditures for each gambling behavior; these were summed together to determine the total money spent on gambling on an annual basis on all gambling behaviors for each participant. The second measure of gambling intensity was overall frequency of gambling. Participants were asked about their frequency of participation for each gambling behavior, selecting one of the six categories. The summary measure of gambling involvement was overall frequency of gambling as measured by the maximum frequency reported for any type of gambling in the past year. A reported frequency of 4 or more times per week (mean 5.5 days/week) was converted to an annual frequency of 286 days (52 weeks × 5.5); 2–3 times per week was given a value of 130 days (52 weeks × 2.5); once a week was given a value of 52 days (52 weeks × 1); 2–3 times per month was given a value of 30 days (12 months × 2.5); and a frequency of less than once a month was given a value of 6 days (12 months × 0.5).

### Analyses

To assess whether problem gambling is more related to some gambling formats (H1), we identified the prevalence of problem gambling among regular gamblers in specific gambling formats using 95% confidence intervals. To examine whether problem gambling is positively related to high involvement in gambling (H2), we examined the Spearman’s correlation between the number of gambling formats an individual engaged in and the individual’s PPGM score. A ROC analysis was also used to assess the relationship between involvement and problem gambling. Finally, using a Mann-Whitney U-test based on 95% confidence intervals, we examined the relationship between problem gambling and number of gambling formats in which an individual participates.

To assess the relationship between involvement in gambling and intensity of gambling (H3), we examined the Spearman’s correlation and used the Fischer’s z-transformation at 95% confidence intervals. To assess whether gambling formats mediate the relationship between involvement and problem gambling (H4), we plotted the prevalence of problem gambling for each form of gambling across increasing numbers of gambling formats. This approach mirrors the Swedish analysis performed by Binde, Romild, and Volberg [5] and is similar to Currie et al.’s [28] examination of gambling frequency among Canadian gamblers.

## Results

### Problem gambling is more closely related to some gambling formats

**Fig. 1:**
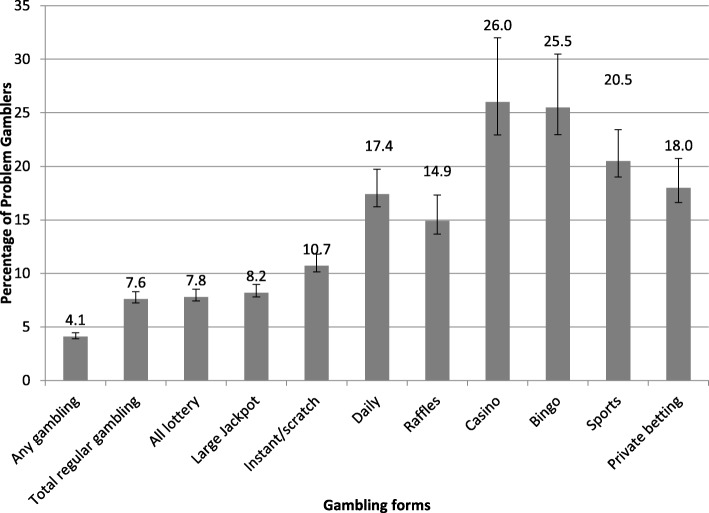
Percentage of problem gamblers among different gambling formats

Depending on the gambling format, the proportion of individuals experiencing a gambling problem varied (Fig. 1). The highest proportions (ranging from 17.4 to 26.0%) of individuals experiencing a gambling problem were among those who gambled regularly (monthly or more often) on casino games, bingo, sports betting, private betting, and daily lottery games. Those who gambled on casino games were more than three times as likely to be classified as problem gamblers compared to those who gambled on all lottery products. The gambling formats that had the lowest proportion of individuals experiencing a gambling problem were all lottery, large jackpot lottery, and instant/scratch tickets, ranging from 7.6 to 10.7%. These findings support Hypothesis 1 that problem gambling is more closely tied to certain gambling formats.

### Problem gambling is positively related to high involvement in gambling

The median number of gambling formats engaged in once a month or more was 2.0 while the mean was similar at 1.97 (Table 1). The number of gambling formats an individual engaged in regularly had a correlation of 0.39 for PPGM-classified problem gamblers. This suggests that regular involvement in multiple gambling formats was positively related to problem gambling. Via the ROC analyses, we find high predictive power between gambling involvement and problem gambling status. The number of gambling formats explained approximately 73% of the variation in whether an individual was experiencing a gambling problem versus not experiencing a gambling problem.

**Table 1:** Gambling involvement in major gambling formats

Min	1
Max	8
Median	2.00
Mean	1.97
Standard Deviation	1.18
Spearman’s Correlation vs. PPGM	0.39*
Area, ROC (PPGM)	0.73

*p < 0.001

**Fig. 2:**
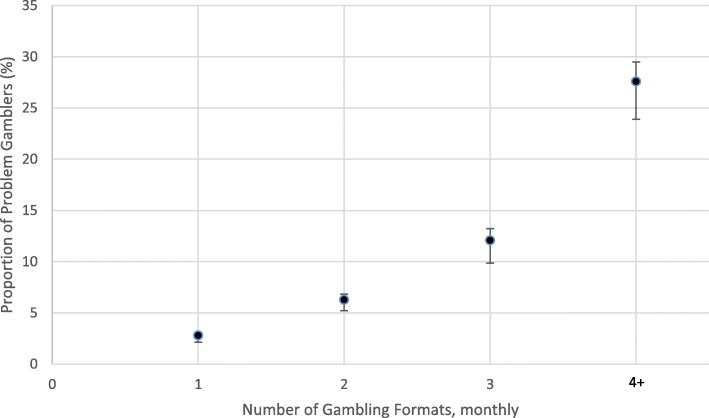
Proportion of problem gamblers relative to number of gambling formats

In our sample of regular gamblers, the overall proportion of PPGM-designated problem gamblers was 7.62% (95%, CI 6.97–8.33). Figure 2 shows that the proportion of regular gamblers experiencing a gambling problem linearly increases as the number of monthly gambling formats increases. There were three times as many individuals experiencing a gambling problem among those who participated in four or more gambling formats andabout 1.5 times as many among those who participated in three gambling formats.

**Fig. 3:**
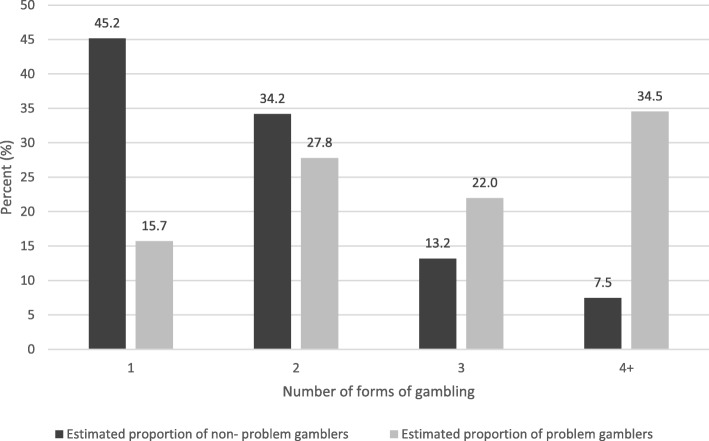
Percentage of non-problem and problem gamblers relative to number of gambling formats

Figure 3 illustrates the overall percentage of individuals experiencing a gambling problem and those not experiencing a gambling problem across number of gambling formats. Among individuals not experiencing a gambling problem, 45% gamble on only one format, while among individuals experiencing a gambling problem, 16% gamble on only one format. Among individuals not experiencing a gambling problem, as the number of monthly gambling formats increases, the proportion decreases, with less than 8% participating in four or more gambling formats on a monthly basis. Among individuals experiencing a gambling problem, as the number of gambling formats increases, the proportion increases, with 34.5% participating in four or more gambling formats on a monthly basis. However, 43.5% of people experiencing a gambling problem participate in only one or two gambling formats.

### Gambling involvement is positively related to gambling intensity

Table 2 examines the relationship between regular gambling involvement and intensity of gambling estimated by money spent on gambling and maximum frequency over the past year (as a proxy for time spent gambling). Examining the relationship between number of gambling formats engaged in regularly (monthly) and money spent in the past year on gambling, the relationship was weak (− 0.20, 95% CI, -0.22, − 0.17). The relationship between frequency of gambling in the past year and involvement was moderate (0.40, 95% CI, 0.38–0.43). While not strong, these results suggest that gambling involvement is positively related to gambling intensity.

**Table 2:** Correlation between gambling involvement and gambling intensity

	Number of major gambling forms at least monthly in past year	Money spent on gambling in past year	Maximum gambling frequency in the past year
Number of major gambling forms at least monthly in past year	–		
Money spent on gambling in past year	−0.20 (−.22, −.17) *n* = 5837	–	
Maximum gambling frequency in the past year	.40 (.38–.43) *n* = 5852	−0.19 (−.21, −.16) *n* = 5837	–

Note: Spearman’s correlation with 95% confidence intervals

### Gambling format mediates the relationship between involvement and problem gambling

**Fig. 4:**
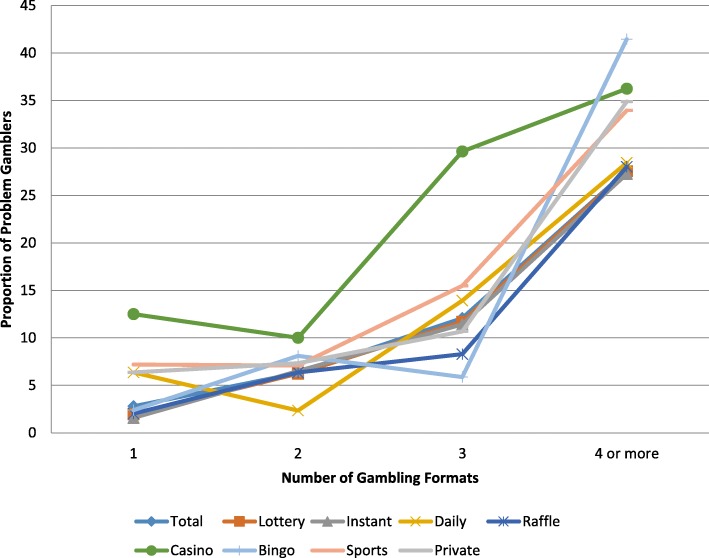
Proportion of problem gamblers relative to gambling format and gambling involvement

Figure 4 illustrates the proportion of individuals experiencing a gambling problem among those who regularly gamble on a specific gambling format. These individuals are categorized within groups of increasing involvement. For example, the first point on the “Casino” line represents those who gambled solely on casino games. The second data point represents those who gambled on casino games regularly and regularly participated in one other gambling format. The third data point contains those who gambled regularly on casino games and participated in two other gambling formats on a monthly basis, etc. As a result, individuals may belong to multiple plotted trends.

Figure 4 demonstrates that regular participation in specific gambling formats may mediate the relationship between involvement and proportion of individuals experiencing a gambling problem. Among the monthly gambling formats across levels of involvement, regular casino gambling was most clearly related to problem gambling with the highest proportion of individuals experiencing a gambling problem (between 10.0–36.2%). Among all regular casino gamblers, 19.6% gambled only on casino games while 14.7% gambled on casino games and one other format (primarily traditional lottery games), 26.5% gambled on casino games and two other formats (primarily traditional and instant lottery games), and 39.2% gambled on casino games and three or more other formats.

Figure 4 also shows that regular bingo participation had the highest proportion of individuals experiencing a gambling problem among those who participated in four or more forms of gambling on a monthly basis (41.5%). Except for those who participated in casino gambling and sports betting, the proportion of individuals experiencing a gambling problem for all other gambling formats was below average (12.1%) among those participating in three gambling formats on a monthly basis. These results confirm Hypothesis 4 that specific gambling formats mediate the relationship between gambling involvement and problem gambling.

## Discussion

The proportion of individuals experiencing a gambling problem was higher among some gambling formats. This supports the first hypothesis. In Massachusetts, regular participation in betting on casino games, bingo, and sports were especially associated with problem gambling. The importance of these formats relative to experiencing gambling problems in Massachusetts is comparable to a similar study conducted by Binde, Romild, and Volberg [5] of gambling behavior in Sweden, which found that regular participation in EGM gambling, casino table games, poker, and bingo was strongly associated with problem gambling. It is interesting that in both Sweden and Massachusetts, casino gambling and bingo were identified as closely related to problem gambling. However, in Massachusetts, unlike Sweden, sports betting also had a higher proportion of people experiencing a gambling problem. Such differences highlight the importance of context since the relationship between a specific gambling format and problem gambling is not static, but dynamic. These relationships are contingent on jurisdictional differences in availability, regulation surrounding the structural characteristics of the formats and their marketing, and socio-cultural differences that influence the uptake and the value placed on specific gambling formats.

High gambling involvement was also found to be positively associated with problem gambling. This supports the second hypothesis. The ROC analysis showed a stronger association between problem gambling and involvement than the Spearman’s correlation test. This suggests that involvement was more strongly associated with whether or not an individual experienced a gambling problem rather than with differences in PPGM scores. This analysis also demonstrates that approximately 16% of individuals experiencing problem gambling participated regularly in only one form of gambling, 28% participated in two forms, 22% participated in three forms, and 35% participated in four or more forms. This supports previous research showing that problem gamblers are more likely to participate in multiple forms of gambling compared to non-problem gamblers [7]. Nonetheless, the average number of formats that problem gamblers regularly participated was 1.97 (median = 2). While these results do support the hypothesis that high involvement in gambling is associated with problem gambling, it is with the large caveat that 43.5% of regular gamblers experienced problems when engaging with only one or two gambling formats.

Gambling involvement was positively associated with intensity of gambling measured in money and frequency (as a proxy for time). This finding supports the third hypothesis. Money and frequency of gambling were associated with regular gambling involvement. Following Binde, Romild, and Volberg’s [5] analysis, there is reason to believe that intensity—which is a defining characteristic of problem gambling—may be partly driving the relationship between involvement and problem gambling.

We also found that the type of gambling format mediated the relationship between involvement and problem gambling. This supports our fourth hypothesis. At all levels of gambling involvement, problem gambling was especially related to regular participation in casino games. Casino gambling had the highest proportion of individuals experiencing a gambling problem across all levels of gambling. The proportion of individuals experiencing a gambling problem who participated in casino gambling ranged from 12.5% for those participating solely in casino gambling to 36.2% of those participating in four or more formats. Our findings support previous studies that suggest that casino gambling (EGMs and table games) may be an especially risky type of gambling [29].

### Limitations

These analyses utilize cross-sectional data, which restricts causal inference. To explicate the temporal sequence between problem gambling, gambling formats, and gambling involvement, longitudinal data is required. Without longitudinal data, we are unable to determine whether participating in a gambling format increases the risk of experiencing a gambling problem or if those who already have a gambling problem are attracted to specific gambling formats. In addition, longitudinal data is needed to understand whether high involvement is a precursor to or simply a symptom of problem gambling. This data also does not distinguish gambling formats based on whether such participation was done at a brick and mortar venue or online. These different forms of access may mediate the relationship between gambling format and problem gambling. In addition, despite utilizing two large datasets, some categorization groupings were quite small leading to estimates that contain large confidence intervals.

## Conclusion

These analyses demonstrate that gambling format is related to whether an individual is likely to experience a gambling problem. We also find that the level of involvement (and its relationship to intensity) may affect the likelihood that an individual will experience problem gambling. Ultimately, however, it appears that the type of gambling format an individual engages in may mediate the relationship between problem gambling and intensity. In the Massachusetts context, participating in casino gambling is more closely associated with problem gambling than other formats.

When comparing these findings to similar analyses [5] and to other studies assessing the relationship between problem gambling and specific gambling formats [13], the consistent finding that casino gambling (particularly EGMs) may be an especially problematic gambling format comes to the fore. In the case of Massachusetts, prior to the opening of land-based casinos in the state, we find that out-of-state casino gambling is especially related to experiences of problem gambling for adult Massachusetts residents. Now that three casinos have opened in Massachusetts (as of June 2019)—increasing the availability of casino gambling to residents—we look forward to examining whether and how the relationships between these axes have changed. In addition, although the results of the present study indicate that involvement in specific forms of gambling is related to problem gambling, further research is needed to explore the significance of this relationship when taking into account other factors such as age, race, gender, socioeconomic status, etc. Nevertheless, this study has found that casino gambling is especially problematic. As a consequence, gambling policy and regulation as well as problem gambling services should focus efforts on casino gambling as a format and environment where individuals may be especially at risk of experiencing gambling problems.

## Data Availability

This manuscript draws on data from the Baseline General Population Survey (BGPS) and the Baseline Online Panel Survey (BOPS) conducted by the Social and Economic Impacts of Gambling in Massachusetts research team based in the School of Public Health and Health Sciences at the University of Massachusetts Amherst. This research is funded by the Massachusetts Gaming Commission. The data are publicly available to other researchers through the Massachusetts Gaming Commission’s Data Storage and Access project and by request to the Massachusetts Gaming Commission, Director of Research and Responsible Gaming, Mark Vander Linden, mark.vanderlinden@state.ma.us.

## Abbreviations

BGPS: Baseline General Population Survey
BOPS: Baseline Online Population Survey
PPGM: Problem and Pathological Gambling Measure
